# A New High Penetrant Intronic Pathogenic Variant Related to Long QT Syndrome Type 2

**DOI:** 10.3390/jcm14134646

**Published:** 2025-07-01

**Authors:** Manuel Rodríguez-Junquera, Alberto Alén, Francisco González-Urbistondo, José Julián Rodríguez-Reguero, Bárbara Fernández, Rut Álvarez-Velasco, Daniel Vazquez-Coto, Lorena M. Vega-Prado, Pablo Avanzas, Eliecer Coto, Juan Gómez, Rebeca Lorca

**Affiliations:** 1 Hospital Álvarez-Buylla, 33611 Mieres, Spain; 2 Área del Corazón, Hospital Universitario Central Asturias, 33011 Oviedo, Spain; 3 Instituto de Investigación Sanitaria del Principado de Asturias, ISPA, 33011 Oviedo, Spain; 4 Unidad de Cardiopatías Familiares, Área del Corazón, Pediatría y Departamento de Genética Molecular, Hospital Universitario Central Asturias, 33011 Oviedo, Spain; 5 Departamento de Medicina, Universidad de Oviedo, 33003 Oviedo, Spain; 6 Centro de Investigación Biomédica en Red de Enfermedades Cardiovasculares (CIBERCV), 28029 Madrid, Spain; 7 Redes de Investigación Cooperativa Orientadas a Resultados en Salud (RICORs), 28029 Madrid, Spain; 8 CIBER-Enfermedades Respiratorias, 28029 Madrid, Spain; 9 Departamento de Morfología y Biología Celular, Universidad de Oviedo, 33003 Oviedo, Spain

**Keywords:** Long QT Syndrome (LQTS), KCNH2, channelopathy

## Abstract

**Background/Objectives**: Long QT Syndrome type 2 (LQT2) is a cardiac channelopathy linked to pathogenic variants in the *KCNH2* gene, which encodes the Kv11.1 potassium channel, essential for cardiac repolarization. Variants affecting splice sites disrupt potassium ion flow, prolong QT interval, and increase the risk of arrhythmias and sudden cardiac death (SCD). Understanding genotype–phenotype correlations is key, given the variability of clinical manifestations even within families sharing the same variant. We aimed to evaluate new pathogenic variants by analyzing genotype–phenotype correlations in informative families. **Methods**: Genetic and clinical assessments were performed on index cases and family members carrying *KCNH2* pathogenic variants, referred for genetic testing between 2010 and June 2023. The next-generation sequencing (NGS) of 210 cardiovascular-related genes was conducted. Clinical data, including demographic details, family history, arrhythmic events, electrocardiographic parameters, and treatments, were collected. **Results**: Among 390 patients (152 probands) tested for LQTS, only 2 *KCNH2* variants had over 5 carriers. The detailed clinical information of 22 carriers of this *KCNH2* p.Ser261fs. has already been reported by our research group. Moreover, we identified 12 carriers of the *KCNH2* c.77-2del variant, predicted to disrupt a splice site and not previously reported. Segregation analysis showed its high penetrance, supporting its classification as pathogenic. **Conclusions**: The newly identified *KCNH2* c.77-2del variant is a pathogenic, as strongly supported by the segregation analysis. Our findings underscore the importance of further research into splice site variants to enhance clinical management and genetic counseling for affected families.

## 1. Introduction

Long QT Syndrome type 2 (LQT2) is a cardiac channelopathy commonly associated with pathogenic variants in the KCNH2 gene, which encodes the α-subunit of the voltage-gated potassium channel (Kv11.1). This channel is crucial for cardiac repolarization. Pathogenic variants result in defective potassium ion flow, impairing cardiac repolarization, and leading to prolonged QT intervals, which can cause arrhythmias and sudden cardiac death (SCD) [1,2]. Among these pathogenic variants, splice site alterations are of particular interest due to their potential to disrupt channel function. The accurate classification of genetic variants is of vital importance, as LQT2 may present as a concealed disease.

A critical aspect of managing LQT2 involves understanding the genotype–phenotype correlation, as the clinical manifestations often exhibit variability, even within families harboring the same pathogenic variant. This variability is further complicated by incomplete penetrance and the influence of environmental factors [3]. Therefore, reaching a definite classification of the pathogenicity is crucial. Precise genotyping allows for better risk stratification and management. In this regard, according to clinical practice guidelines, all carriers of pathogenic variants in LQT2 should receive treatment with beta-blockers (BBs) to prevent life-threatening arrhythmic events [4,5].

Recent studies have highlighted the importance of genetic testing and clinical assessment to guide therapy decisions and predict arrhythmic risk in LQT2 patients [6,7]. Novel pathogenic variants have been described with NGS techniques, understanding their clinical impact [8]. This study aims to analyze novel pathogenic variants of LQT2, focusing on their penetrance and expressivity within affected families and their correlation with their clinical outcomes.

The objectives are to identify an informative cohort of patients carrying novel pathogenic variants of Long QT Syndrome Type 2 (LQTS2); to analyze potential differences in penetrance and expressivity among family members carrying the same pathogenic variant of the channelopathy; and to assess the genotype–phenotype correlation.

## 2. Materials and Methods

### 2.1. Study Population

A retrospective review was conducted on all patients referred for genetic testing between 2010 and June 2023. All consecutive index cases diagnosed with LQTS and referred for genetic testing were identified. The following selection criteria were applied:

Inclusion criteria:

1. Patients under clinical follow-up in the Principality of Asturias.
2. Carriers of pathogenic variants in the *KCNH2* gene.
3. Informative families, with at least 5 carriers evaluated.

Exclusion criteria:

1. Patients referred from other regions only for genetic testing without clinical follow-up in the Principality of Asturias.
2. Variants identified in fewer than six carriers within the cohort.
3. Variants of uncertain significance or benign variants in *KCNH2*.

### 2.2. Phenotypic Evaluation

All available patients underwent genetic and clinical assessments. Clinical data were recorded in a customized registry, including demographic information, personal and family history of symptoms, arrhythmic events, electrocardiographic parameters, and therapies.

Electrocardiographic parameters were measured following the methodology established by the International Long QT Syndrome Registry. The first available 12-lead electrocardiogram (ECG) was obtained (paper speed: 25 mm/s, voltage setting: 10 mm/mV) before therapy initiation, whenever possible, at stable heart rates close to 60 bpm during daytime to minimize the confounding effect of diurnal QT interval variability. The QT interval was measured in lead II or V5 and corrected for heart rate using Bazett’s formula.

LQTS penetrance was calculated as the percentage of carriers of a pathogenic variant exhibiting a positive phenotype, defined as a prolonged QTc interval (>440 ms in males and >460 ms in females). Two obligate carriers who died without clinical evaluation or available ECGs were excluded from penetrance calculations due to unknown clinical status. Pathogenic variant carriers (genotype-positive) were categorized into three groups: (a) phenotype-positive carriers with prolonged QTc and cardiac-related symptoms (arrhythmia, syncope, aborted sudden death, or sudden death); (b) phenotype-positive carriers with prolonged QTc but no cardiac-related symptoms; and (c) phenotype-negative carriers with a normal QTc and no cardiac-related symptoms.

Phenotype positivity was determined based on QTc values in the studied individuals, with QTc prolongation defined as >440 ms in males and >460 ms in females (measured in lead V5 and corrected using Bazett’s formula). Penetrance was calculated as the proportion of individuals carrying a pathogenic KCNH2 variant (genotype-positive for LQTS) who also exhibited the associated trait (phenotype-positive). A phenotype was considered positive if the carrier presented with a prolonged QT interval on ECG or if an obligate carrier had suffered sudden death at a young age without prior clinical evaluation.

Clinical and demographic data were reviewed, including personal and family history of symptoms, arrhythmic events, ECG parameters, device implantation, and medical therapies (see Supplementary Material, Table S1). Echocardiography was not systematically performed in family screening, as LQTS is a channelopathy. Records from implanted automatic defibrillators (ICDs) were also reviewed.

All patients provided written informed consent for the use of their genetic data for research purposes. The study protocol adhered to institutional ethical guidelines and was approved by the local Ethics Committee (CeimPA 2025.109).

### 2.3. Genetic Testing Procedure

Blood samples were collected from all patients who consented to genetic testing using 9 mL EDTA anticoagulant tubes. DNA was extracted from peripheral blood leukocytes using the standard salt extraction method, a simple and non-toxic technique yielding high-quality DNA.

Genetic testing was performed on DNA samples from all referred patients. Index cases with LQTS underwent next-generation sequencing (NGS) targeting 210 genes associated with cardiovascular diseases, including the LQTS-related gene. These genes were sequenced using Ion Torrent technology, which employs semiconductor chips and an Ion GeneStudio S5 sequencer (Thermo Fisher Scientific, Waltham, MA, USA). The detailed procedure has been previously described.

Raw data were processed using Torrent Suite v5 software. Read assembly and variant identification were performed with Variant Caller (VC). Variant annotation, including population, functional, disease-related, and in silico predictive algorithms, was conducted using Ion Reporter (Thermo Fisher Scientific) and HD Genome One (DREAMgenics SL, Oviedo, Spain). The Integrative Genome Viewer (IGV, Broad Institute, Cambridge, MA, USA) was used for in-depth coverage analysis, sequence quality assessment, and variant identification.

Familial variant screening was conducted via Sanger sequencing using an ABI3130XL sequencer (Thermo Fisher Scientific). The interpretation of all genetic variants with an allele frequency < 0.01 followed the 2015 American College of Medical Genetics and Genomics (ACMG-AMP) standards and guidelines [9].

### 2.4. Statistical Analysis

Statistical analyses were performed using SPSS v.25 (SPSS Inc., Chicago, IL, USA). Descriptive statistics for continuous variables were presented as mean ± standard deviation (SD), while categorical variables were expressed as frequencies or percentages. Frequency comparisons were conducted using the chi-square test or Fisher’s exact test, with statistical significance set at *p* < 0.05.

## 3. Results

**Figure 1:**
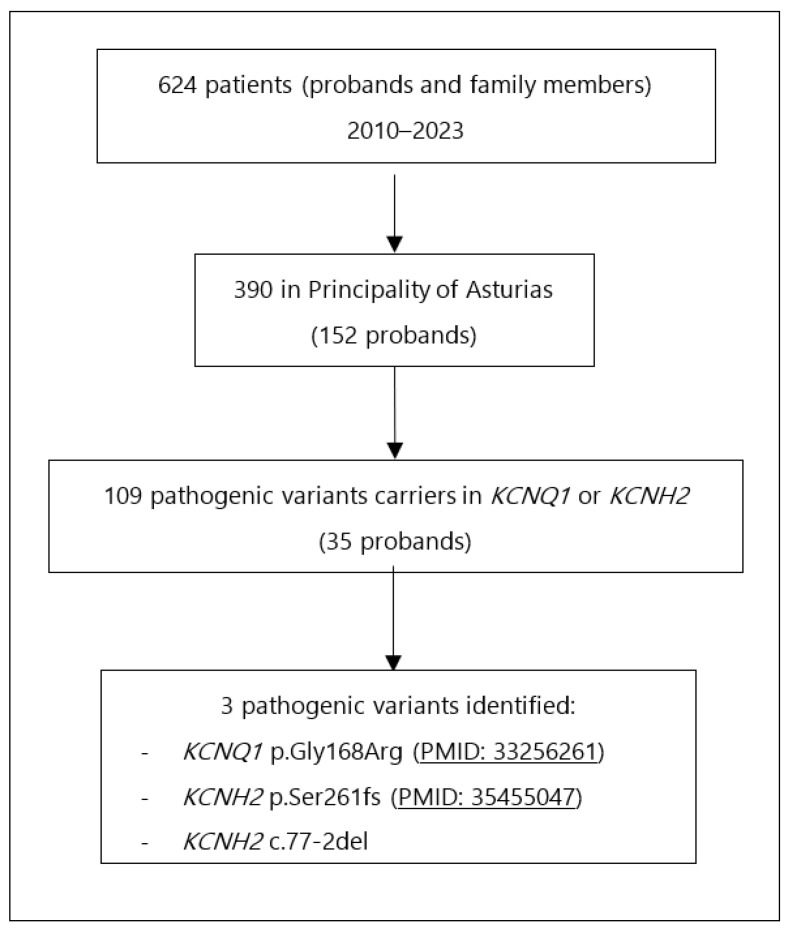
Flowchart for carriers’ identification.

A review was conducted on all index cases and family members referred for genetic testing between 2010 and June 2023. Only patients from the Principality of Asturias were included in this study. Among 390 patients (152 probands), 109 were carriers of pathogenic variants in *KCNQ1* or *KCNH2* (35 probands). Two pathogenic variants in *KCNH2* and one in KCNQ1 were identified with more than five carriers (Figure 1). All patients identified with pathogenic variants of LQTS Type 2 were included in the study.

### 3.1. Carriers of the Pathogenic Variant KCNH2 p.Ser261fs

The detailed clinical information of all 22 carriers of this variant has already been reported by our research group [10]. This was the first time this variant has been described in the literature. The penetrance was 100% and presence of malignant events among this LQTS cohort was highly significant. No significant differences were found between sexes in either penetrance (100% in both genders) or potentially lethal cardiac events. QTc duration also showed no statistical differences between sexes [10].

### 3.2. Carriers of the Pathogenic Variant KCNH2 c.77-2 del

**Figure 2:**
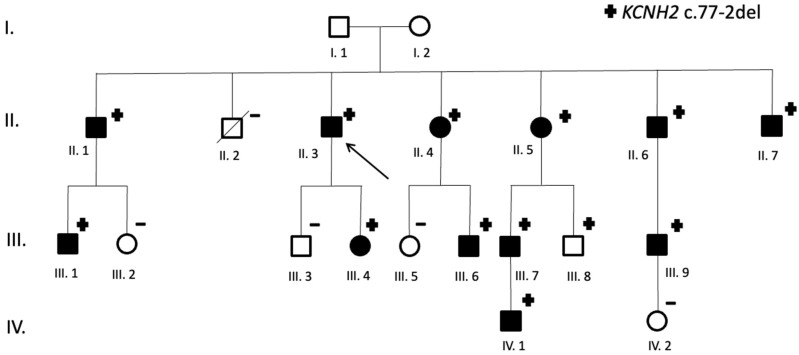
Family pedigree (*KCNH2* c.77-2del carriers). Symbols denote sex and disease status: +, carriers; −, noncarriers; without a sign, genetic status unknown; box, male; circle, female; box/circle black darkened, LQTS phenotype; box/circle clear, negative phenotype; slashed, deceased; arrow, proband.

The proband was a male patient referred after a long QTc was identified in a routinary ECG. Family screening identified another 12 carriers of *KCNH2* c.77-2 del: 9 men and 3 women (Figure 2). Most evaluated patients (12/13) presented prolonged QTc in the ECG. Therefore, penetrance is 92.3%. While all identified women carriers presented at least 1 ECG with prolong QTc, one male carrier had all registered ECGs with normal QTc intervals to date.

An implantable cardioverter–defibrillator had been indicated in two patients, one of them due to repeated syncope and non-sustained ventricular tachycardia despite optimal medical treatment.

**Figure 3:**
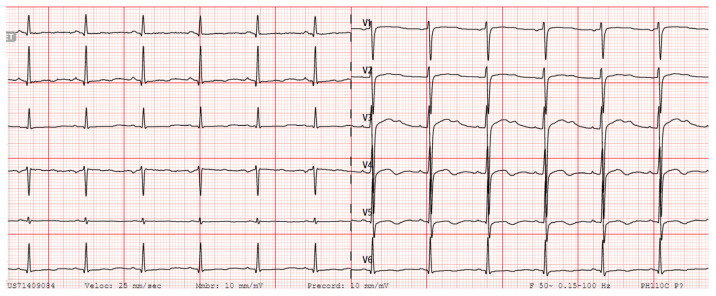
Patient’s II.7 ECG, in sinus rhythm at 79 beats per minute, with a prolonged QTc interval of 528 milliseconds and notched T wave.

The clinical data of all identified carriers’ of *KCNH2* c.77-2del variant are summarized in Supplementary Table S1. A representative ECG with typical *KCNH2*- QTc prolongation is displayed in Figure 3 (patient II.7).

The sequence change *KCNH2* c.77-2del falls at the -2 position of intron 1 of the *KCNH2* gene. This sequence change affects a donor splice site in intron 1 of the gene. Although functional studies have not been reported, algorithms developed to predict the effect of sequence changes on RNA splicing suggest that this variant would disrupt the consensus splice site, resulting in an aberrant mRNA. The putative effect of the intronic variant on RNA splicing can be determined using bioinformatics programs such as the BDGP Splice Site Predictor (https://www.fruitfly.org/seq_tools/splice.html (accesed on 5 May 2025)). Variants that disrupt the donor or acceptor splice site typically led to a loss of protein function [11], and loss-of-function variants in *KCNH2* are known to be pathogenic [12,13]. Moreover, this variant is not present in population databases (ExAC no frequency) and has never been reported in the literature in individuals with *KCNH2*-related conditions. ClinVar does not contain an entry for this variant either. In addition, segregation data strongly support the variant’s pathogenicity. According to ACMG/AMP guidelines [9], the combination of 1 Strong (PP1), 2 Moderate (PVS1, PM2), and 1 Supporting (PP3) criteria is sufficient to classify the variant as pathogenic (Table 1). These revisions align with ClinGen SVI recommendations and justify the pathogenic classification based on the combination of 1 Strong, 2 Moderate, and 1 Supporting criteria [9].

## 4. Discussion

The identification of the *KCNH2* c.77-2 del variant in our cohort represents a significant contribution to the understanding of genetic variations associated with LQT2. Variants in the *KCNH2* gene, particularly those that lead to a loss of function, are known to reduce the repolarizing potassium current (IKr), thereby prolonging the action potential duration and increasing the risk of arrhythmic events [1,2]. While pathogenic variants in *KCNH2* are well-established as pathogenic for LQT2, the phenotypic expression can differ significantly, even among family members carrying the same genetic change. This variability may be attributed to factors such as gender differences, environmental influences, and potential epigenetic modifications [3]. In our study, the penetrance of both pathogenic variants and *KCNH2* c.77-2del variant carriers was high and not sex-dependent. The *KCNH2* c.77-2del change has never been reported in the literature. However, according to the data presented in Section 3, it can be classified as pathogenic. Other nearby changes and their classification reported in ClinVar can be consulted in Table 1. For instance, whereas *KCNH2* c.77-1G > A or c.77-1G > T are classified as likely pathogenic, *KCNH2* c.77-3del and c.77-3_77-2dup are considered VUS, and *KCNH2* c.77-3T > C and c.77-4C > T are classified as likely benign (Table 2).

LQTS is an autosomal dominant inherited condition that can present with syncope, palpitations associated with premature ventricular beats or sudden cardiac death. Clinical manifestations are more frequent in youth. Although the primary genes associated with LQTS were identified many years ago, understanding the description of specific variants and arrhythmic risk remains incomplete. Beta-blockers are still the main treatment for LQTS. Nonselective beta-blockers and especially propranolol and nadolol are considered first-line therapy, with a demonstrated reduction in malignant events (syncope, aborted or fatal SCD) in all age ranges [14]. Device therapy should be reserved for those symptomatic patients despite beta-blocker therapy [15,16,17]. Other therapies may be considered such as other medications, like mexiletine, that has been shown to be useful to shorten the QT interval [18,19] or left cardiac sympathetic denervation (LCSD) [20,21]. In this context, precision medicine represents a major challenge in current clinical practice. In this context, advancements in genetics are significantly contributing to this clinical objective. This is particularly notable in inherited diseases, such as channelopathies, including LQTS [22]. Regarding therapeutic considerations in this cohort, carriers of the c.77-2del variant were all treated with beta-blocker therapy [14], showing variable responses to beta-blocker therapy, with some degree of QTc shortening observed, though not uniformly. No patients in this family received additional treatments such as mexiletine or left cardiac sympathetic denervation (LCSD). Importantly, splice site variants like c.77-2del may offer promising targets for emerging antisense-based therapies. In particular, splice-switching oligonucleotides (SSOs) have been explored in other genetic disorders to modulate splicing patterns and restore normal transcript processing [23,24,25,26]. The clinical success of SSO therapies in other monogenic diseases (e.g., spinal muscular atrophy, Duchenne muscular dystrophy) further supports their translational potential in LQTS [25,27]. Currently, SSO-based approaches for LQTS remain experimental. Proof-of-concept studies in LQTS models, particularly for KCNH2 (LQT2), have demonstrated that antisense oligonucleotides can modulate splicing and increase functional channel expression, drawing on lessons from cystic fibrosis and other channelopathies. However, no SSO therapy for LQTS has reached clinical trials as of 2025 [28,29,30]; the identification of well-characterized disease-causing splice site pathogenic variants could provide a rationale for its future development and application.

Loss-of-function variants in the *KCNH2* gene cause potassium channel disfunction, leading to a prolonged ventricular action potential duration, the basis of LQTS [31,32]. More than half of the pathogenic variants described in *KCNH2* are nonsense [33,34]. Individualized risk stratification is crucial. QTc duration has been regarded as the strongest predictor [35], although a correlation between QTc and clinical events has not been found. It depends on the affected gene and specific variant [31,36]. Accordingly, numerous studies and consensus documents have emphasized the relevance of the genotype in arrhythmic risk prediction [31,37,38,39,40]. Patients with LQT2 and LQT3 are generally exposed to a greater risk of life-threatening events than those with LQT1 [36,41,42]. Multiple studies have explored genotype–phenotype correlations by analyzing cohorts of index patients carrying pathogenic variants within the same gene. However, this channelopathy exhibits incomplete penetrance and variable expressivity [16,31], making it difficult to establish definitive genotype–phenotype associations for arrhythmic risk. As a result, investigating large families with an identical pathogenic variant may offer crucial insights into this complex relationship.

Splice site variants are a well-established pathogenic mechanism in several LQTS-associated genes, including KCNQ1, KCNH2, and SCN5A. In KCNQ1, splice site mutations such as c.1032G > A and c.922-1G > C have been shown to cause exon skipping or the activation of cryptic splice sites, resulting in truncated or dysfunctional Kv7.1 channels and a loss-of-function phenotype, paralleling the effects seen in KCNH2 variants. Functional studies confirm that these aberrant transcripts produce non-functional channels and can exert dominant-negative effects by trapping wild-type proteins intracellularly [43,44,45,46].

Similarly, splice site variants in SCN5A have been identified in LQTS patients, with evidence that such mutations disrupt normal splicing and contribute to arrhythmogenic phenotypes, including LQTS type 3 [27]. In KCNH2, splice site mutations can lead to aberrant splicing, such as intron retention or cryptic splice site activation, resulting in non-functional channels and LQTS [47,48].

The clinical expressivity and severity of splice site variants are variable and depend on the specific gene, the nature of the splice alteration, and potential genetic modifiers, as demonstrated by studies of synonymous and non-canonical splice variants in KCNQ1 that modulate phenotype severity [49]. These findings underscore the necessity of evaluating splice site variants in the context of gene-specific and patient-specific factors to accurately assess pathogenicity and guide management [43,45,49].

The discovery of the *KCNH2* c.77-2 del variant in our cohort provides new insights into the genetic landscape of LQT2. This specific splice site variant, which occurs two bases before the canonical splice site, leads to a predicted disruption of the normal splicing mechanism, potentially resulting in a truncated or non-functional protein. The pathogenicity of this variant is underscored by its location in a region closely associated with other known pathogenic variants, particularly those at position -1, which are recognized as causing the loss of channel function. In contrast, variants at position -3, which have been previously described, have been classified as variants of uncertain significance (VUS), due to the ambiguity in their functional consequences.

The presence of a pathogenic variant at position -2 in *KCNH2* supports the hypothesis that alterations in this region disrupt the splicing process and subsequently impair potassium channel function. The c.77-2 del variant adds to this understanding, as it is positioned within the same genomic area and exhibits similar molecular consequences. This highlights the potential pathogenic role of the splice site variant in this region, further reinforcing the importance of considering such alterations when assessing genetic risk in LQT2. The classification of variants of uncertain significance (VUSs), such as those at position -3, remains a challenge in genetic diagnostics. These variants are often difficult to interpret due to their unclear impact on gene function, requiring further research and larger patient cohorts to establish their clinical relevance. In contrast, the clear pathogenic impact of the KCNH2 c.77-2 del variant provides a valuable contribution to the understanding of splice site mutations in LQT2, offering a more definitive tool for genetic counseling and risk assessment in affected families.

In addition to the novel identification of this variant, our study emphasizes the importance of genotype–phenotype correlation in managing LQT2. While clinical manifestations in LQT2 can vary widely, understanding the specific variant involved is crucial for accurate risk stratification and treatment planning. This is especially true for splice site variants, which can have complex and variable effects depending on the precise location and type of alteration. The identification of the *KCNH2* c.77-2del variant provides a clearer framework for understanding these kinds of variants and underscores the need for continuous research into the genetic underpinnings of LQT2.

In conclusion, the new *KCNH2* c.77-2del variant identified in our cohort is a pathogenic variant that contributes to the growing body of knowledge on splice site alterations in LQT2. Its presence in a family with multiple affected individuals highlights the importance of considering splice site variants in genetic diagnostics for LQT2. By improving our understanding of these pathogenic variants, we can enhance clinical management strategies and provide more accurate genetic counseling for affected families.

Future investigations that incorporate comprehensive epigenomic profiling and functional studies are expected to clarify the mechanisms underlying variable expressivity and incomplete penetrance in LQTS, particularly among carriers of splice site variants such as c.77-2del. Current evidence demonstrates that, beyond the primary pathogenic variant, additional genetic factors—including rare variants in other ion channel genes, genes involved in cardiac electrophysiology, and established or novel modifier genes—can significantly modulate disease severity and arrhythmic risk. For example, rare variants in cardiac genes outside the primary LQTS gene have been shown to increase the Schwartz score and clinical severity among LQTS patients, supporting a polygenic or oligogenic model of disease expression [50,51,52,53].

Epigenetic mechanisms—such as DNA methylation, histone modifications, and non-coding RNAs—are increasingly recognized as contributors to phenotypic variability in inherited cardiac diseases. Studies in related channelopathies and cardiomyopathies have shown that differential DNA methylation patterns can correlate with disease severity, even among individuals with identical pathogenic variants, suggesting that epigenetic regulation may influence gene expression and cardiac electrophysiology. Although direct evidence in LQTS is still emerging, these findings support the hypothesis that epigenetic modifications could underlie differences in arrhythmic risk and symptom manifestation among carriers of the same splice site mutation [54,55].

Understanding the interplay between primary pathogenic variants, genetic modifiers, and epigenetic factors is critical for the accurate risk stratification and personalized management of LQTS patients. Integrating these data may enable a more precise prediction of arrhythmic risk, inform surveillance strategies, and guide therapeutic decisions, moving beyond a one-size-fits-all approach to individualized care [37,55,56].

## 5. Conclusions

We identified a new pathogenic intronic splice site variant associated with LQTS: *KCNH2* c.77-2del. This variant adds important new information to the body of knowledge surrounding LQT2 and its genotype–phenotype correlations. Our findings highlight the need for further investigation into the implications of splice site variants to improve clinical management and genetic counseling for affected families.

## Figures and Tables

**Table 1:** ACMG/AMP classification of KCNH2 c.77-2del [9].

Code	ACMG/AMP Criterion	Given Strength	Justification
PVS1	Null variant (canonical ± 1 or 2 splice sites) in a gene where a loss of function is a known mechanism of disease	Moderate	The c.77-2del variant affects the canonical splice acceptor site of exon 2. Splicing prediction tools (e.g., BDGP, SpliceAI) suggest exon skipping that would likely result in a frameshift and premature termination codon. However, since no RNA studies are available to confirm aberrant splicing, PVS1 is conservatively applied at moderate strength (PVS1_Moderate) according to ClinGen SVI guidance.
PM2	Absent from population databases	Moderate	The variant is absent from large population databases, supporting its rarity and potential pathogenicity.
PP1	Co-segregation with disease in multiple affected family members	Strong	The variant segregates with disease in at least 10 informative meioses across a multigenerational family, including siblings, cousins, and one affected descendant, supporting PP1_Strong based on ACMG thresholds.
PP3	Multiple lines of computational evidence support a deleterious effect	Supporting	In silico tools consistently predict that the variant disrupts the canonical splice acceptor site, leading to abnormal splicing (e.g., BDGP, SpliceAI), fulfilling PP3.

American College of Medical Genetics and Genomics (ACMG) guidelines criteria: PVS: Very Strong; PS: Strong; PM: Moderate, PP: Supporting criteria [9].

**Table 2:** Single-nucleotide variants reported in the ClinVar database affecting close residues to *KCNH2* c.77-2del (www.ncbi.nlm.nih.gov/clinvar/intro (accessed on 1 February 2025)).

Gene	c.DNA	Protein Change	Allele Frequency (gnomAD)	Molecular Consequence	Interpretation (Number of Submissions)
*KCNH2*	c.80del	p.Arg27fs	Absent	Frameshift	LP (1)
*KCNH2*	c.80G > A	p.Arg27His	0.000001	Missense	VUS (2); LP (1)
*KCNH2*	c.80G > C	p.Arg27Pro	Absent	Missense	-
*KCNH2*	c.77G > T	p.Ser26Ile	Absent	Missense	-
*KCNH2*	c.77-1G > T	-	Absent	splice acceptor	LP (1)
*KCNH2*	c.77-1G > A	-	Absent	splice acceptor	LP (2)
*KCNH2*	c.77-3_77-2dup	-	Absent	splice acceptor	VUS (1)
*KCNH2*	c.77-3T > C	-	Absent	Intron variant	LB (1)
*KCNH2*	c.77-3del	-	Absent	Intron variant	VUS (1)
*KCNH2*	c.77-4C > G	-	0.0000007	Intron variant	VUS (1)
*KCNH2*	c.77-4C > T	-	0.000005	Intron variant	LB (3)

P: pathogenic, LP: likely pathogenic; VUS: variant of unknown significance; LB: likely benign; gnomAD: the Genome Aggregation Database Version: 4.1.

## Data Availability

Data supporting this study are included within the article and/or Supplementary Materials. Additional data are available from the corresponding authors upon reasonable request.

## Supplementary Materials

The following supporting information can be downloaded at https://www.mdpi.com/article/10.3390/jcm14134646/s1, Table S1: Carriers clinical data.

## Abbreviations

The following abbreviations are used in this manuscript:

LQT2	Long QT Syndrome type 2
SCD	Sudden cardiac death
NGS	Next-generation sequencing
