# Supplementary material for: A New High Penetrant Intronic Pathogenic Variant Related to Long QT Syndrome Type 2
Source: J Clin Med. 2025 Jul 1;14(13):4646. doi: 10.3390/jcm14134646 (PMC12251465; doi:10.3390/jcm14134646)
Supplement: Supplementary file 1 [file jcm-14-04646-s001.zip › jcm-3658069-supplementary.pdf]

Table S1. Clinical data of all identified carriers' of KCNH2 c.77-2del variant.

	Gender	Age at diagnosis	Reason for Evaluation/Referral	Symptoms	Arrythmia	Stress test	ICD implantation	Events	Actual treatment	First-longest QTc (QT) msec	Last available QTc (QT) msec
II.1	Male	48	Family screening	Syncope	NSVT	-	Yes (53 yo)	No	Propranolol 10mg t.i.d.	510 (459)	494 (494)
II.3	Male	59	Proband	None	No	-	No	No	None (Asthma)	520 (*)	495 (476)
II.4	Female	54	Family screening	None	No	-	No	No	Bisoprolol 2.5mg q.d.	457 (478)	478 (509)
II.5	Female	57	Family screening	None	No	-	No	No<br>Death (sepsis)	None (Severe cognitive decline and amyotrophic lateral sclerosis)	465 (354)	465 (354)
II.6	Male	59	Family screening	None	No	-	No	No	None (bradycardia at diagnosis)	Prolonged*	440 (496)
II.7	Male	50	Family screening	None	No	-	No	No	None (Severe cognitive decline)	528 (460) Notched T wave	466 (378) Notched T wave
III.1	Male	14	Family screening	None	No	Normal QT shortening	No	No	Bisoprolol 2.5mg q.d.	457 (466)	444 (463)
III.4	Female	-	Family screening	None	No	-	No	No	Unknown^	Prolonged*	Unknown^
III.6	Male	35	Family screening	None	No	Abnormal QT	Yes (44 yo)	No	Propranolol 20mg b.i.d.	563 (*)	550 (587)

						prolongation and VE.			(does not tolerate higher dosis)		
III.7	Male	25	Family screening	None	No	Normal QT shortening.	No	No	Nadolol 40mg q.d.	Unknown^ Notched T wave	442 (398) Notched T wave
III.8	Male	28	Family screening	None	No	Normal QT shortening	No	No	None (bradycardia at diagnosis)	Normal*	411 (433)
III.9	Male	..	Family screening	None	No	Abnormal QT prolongation	No	No	Bisoprolol 2.5mg q.d..	470 (441) Notched T wave	406 (447) Notched T wave
IV.1	Male	1	Family screening	None	No	-	No	No	Propranolol 2.2mg/kg/day	452 (332)	439 (294)

NSVT: non sustained ventricular tachycardia; ICD: implantable cardioverter-defibrillator; VE: ventricular extrasystole; yo: years old; t.i.d: t*er in die*; q.d: q*uaque die*; b.i.d: *bis in die*; QTc: corrected QT interval; msec: milliseconds. *ECG not available to review (noted as prolonged and labelled as LQTS secondarily in old records); ^: followed up in other hospital.
